# Probing motor dynamics at the muscle level—Acoustic myography in Parkinson's disease

**DOI:** 10.14814/phy2.15631

**Published:** 2023-03-22

**Authors:** M. Celicanin, A. P. Harrison, J. Kvistgaard Olsen, L. Korbo, A. Løkkegård, B. Danneskiold‐Samsøe, H. R. Siebner, T. V. Ilic, E. M. Bartels

**Affiliations:** ^1^ Department of Neurology Copenhagen University Hospital, Bispebjerg and Frederiksberg Copenhagen Denmark; ^2^ The Parker Institute Copenhagen University Hospital, Bispebjerg and Frederiksberg Frederiksberg Denmark; ^3^ University of Copenhagen, PAS (Physiology) Faculty of Health and Medical Sciences Kobenhavn Denmark; ^4^ Danish Research Centre for Magnetic Resonance, Centre for Functional and Diagnostic Imaging and Research Copenhagen University Hospital Amager and Hvidovre Hvidovre Denmark; ^5^ Department of Neurophysiology, Medical Faculty of Military Medical Academy University of Defense Belgrade Serbia

**Keywords:** acoustics, muscle tonus, muscles, myography, Parkinson´s disease

## Abstract

Acoustic myography (AMG) noninvasively probes muscle activity. We explored whether AMG captures abnormal motor activity in patients with Parkinson's disease (PD) and how this activity is modulated by antiparkinsonian medication. Twenty patients with PD underwent AMG of the *biceps, triceps, extensor carpi radialis longus, and adductor policis* muscles of the more affected arm during active and passive movements, using a mobile AMG device (CURO, Denmark). AMG and assessment of motor symptoms were performed in a pragmatic off‐medication state, as well as one and 3 h after oral intake of 200 mg levodopa. Three AMG parameters were calculated using the CURO analysis system. Motor efficiency was expressed by the E‐score, muscle fiber recruitment by the temporal T‐score, spatial summation by the S‐score, and S/T ratio. Twenty age‐ and sex‐matched healthy subjects served as controls. Group mean values were statistically compared using unpaired two‐tailed adjusted *t‐*test and ANOVA with Tukey´s correction for multiple comparison (*p* ≤ 0.05). For the biceps and extensor carpi radialis longus muscles, the active movement S:T ratio was lower in PD relative to healthy controls. The E‐score was also lower during active and passive flexion/extension movements in the off‐medication state. No significant between‐group differences in the AMG scores were noted for the triceps muscle during active or passive movements. The active S:T ratio and the E‐score during active elbow flexion and extension may offer a useful means to quickly assess abnormal motor activity and the effect of drug treatment in PD.

## Graphical abstract

Acoustic myography (AMG) is objective, noninvasive, reliable method, which can be used for assessing a muscle condition in Parkinson's patients. Acoustic myography revealed abnormal dynamics of *m. biceps* activity in Parkinson patients. Spatial summation of activity was augmented relative to temporal summation when compared to healthy controls. The ratio between spatial and temporal summation is scaled with total UPDRS score. Activation efficacy was reduced in non‐medicated patients during active and passive movements.

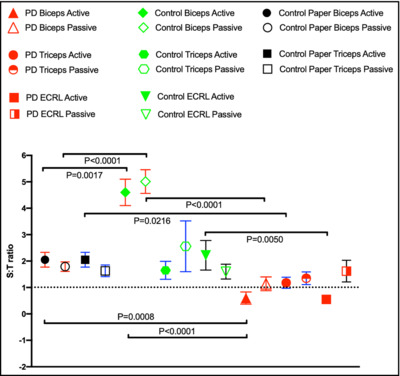

## 1 INTRODUCTION

Parkinson´s disease (PD) is a neurodegenerative disease clinically presented by motor and non‐motor features. The cardinal motor features are slowness of movement (bradykinesia) and tremor, as well as muscular rigidity and postural instability (Tarakad & Jankovic, 2017). An accurate diagnostic workout for PD is still based on clinical assessment which may be supported by neuro‐imaging, since no specific test exists (Massano & Bhatia, 2012; Poewe et al., 2017). Surface electromyography (sEMG) has been used as an objective method to distinguish PD subjects from healthy subjects (Robichaud et al., 2009). Indeed, subjects with PD need between 15 and 20 min for their muscles to relax close to a baseline sEMG signal, while healthy individuals achieve this almost instantly (Cantello, 1995). In PD subjects, muscle rigidity decreases with levodopa medication (McLellan, 1973). In fact, recently it was shown that improvements in resting elbow joint angle follow medication (mainly levodopa or levodopa and dopamine agonists) (Marusiak et al., 2018). These findings also lend support to the idea that rigidity is present to a higher degree in flexors than in extensor muscles and that the tonic stretch reflex, which has been found to increase more in flexor than extensor muscles, affects the resting joint angle in PD subjects (Andrews et al., 1972).

Acoustic myography (AMG) records the pressure waves emitted by contracting muscle fibers with a piezo‐ceramic sensor placed on the skin above the muscle of interest. AMG enables noninvasive, quick, and reliable muscle assessment, and it lends itself to home measurement of subjects performing daily tasks over a number of hours (Claudel et al., 2018; Harrison, 2018; Harrison et al., 2013). Pain assessment was included in this study to determine and exclude possible muscle disease clinically presented by pain (Freynhagen et al., 2006). AMG has the potential to accurately assess muscle fiber activity, thereby indicating precisely how an individual muscle is activated and regulated during any given function. The method has, apart from studies on healthy subjects (Bartels, Ahmed, et al., 2020; Claudel et al., 2018; Freynhagen et al., 2006), successfully been applied in cerebral palsy (CP) subjects, where a different recruitment pattern of muscle fibers was seen (Pingel et al., 2019).

The aim of this study was therefore to explore the potential use of AMG in the motor assessment of PD during active and passive single‐joint movements of the upper limb. We hypothesized that patients with PD would show a reduced level of motor efficiency, presumably caused by slower muscle fiber recruitment and reduced summation of muscle fiber activity.

## 2 MATERIALS AND METHODS

### 2.1 Ethics

The study was conducted in accordance with the guidelines set by the Helsinki Declaration 2013 (https://www.wma.net/policies‐post/wma‐declaration‐of‐helsinki‐ethical‐principles‐for‐medical‐research‐involving‐human‐subjects/). The subjects gave informed written consent prior to participating in the study. Experimental procedures were approved by the Capital Region of Denmark's Ethics Committee (No. H‐17021637), and data collection and handling were registered with and approved by the Danish Data Protection Agency. All data were handled according to The Act on Processing of Personal Data (Act No. 429 of May 31, 2000, with amendments [latest 2018]) implementing Directive 95/46/EC on the protection of individuals with regard to the processing of personal data and on the free movement of such data.

### 2.2 Subjects

Twenty patients fulfilling the diagnostic criteria for PD referring to UK Parkinson disease Society Brain Bank Criteria (Berardelli et al., 2013) were recruited at the Department of Neurology, Copenhagen University Hospital, Bispebjerg and Frederiksberg, Denmark. Only patients with a disease duration of <10 years and a Montreal Cognitive assessment (MoCa) (Rossetti et al., 2011) score above 25 were included. Patients also needed to be able to understand instructions in Danish and to answer questionnaires in Danish. All patients received dopamine replacement therapy and needed to be capable to pause antiparkinsonian medication for 12 h. Patients with levodopa‐induced dyskinesia, or Levodopa and apomorphine infusion pump treatment or deep brain stimulation (DBS) were not included.

The inclusion criteria for the patients were as follows: Parkinson's disease; the Montreal Cognitive assessment (MoCa) (Rossetti et al., 2011) >25; duration of disease <10 years; ability to understand instructions in Danish and to answer questionnaires in Danish; mono/multi treated with PD drugs; and capable of being free of PD medicine for 12 h.

### 2.3 Healthy subjects

Twenty age‐ and sex‐matched healthy subjects were recruited.

The inclusion criteria for healthy subjects were as follows: 18.5 < BMI < 30; healthy according to an examination by a physician (MC); reporting of no chronic neuromuscular or movement disorder; no earlier history of affection of the nervous system or no present illness; pain reported in the normal range and pattern for healthy subjects from answering the PainDETECT Questionnaire (PD‐Q) ©2005 Pfizer Pharma GmbH (Freynhagen et al., 2006) prior to participation and ability to understand instructions in Danish and to answer questionnaires in Danish.

### 2.4 Measurement schedule

Patients were selected during consultations in an outpatient clinic, and the study was explained in detail with possibility of asking further questions. All patients had then a minimum of 1 day to consider their participation in the study. If participation was confirmed by mail or during a phone call, PD patients were visited at their home the day before AMG measurements were performed. The study aims and procedures were again explained to the patients who had the possibility of asking further questions prior to signing the informed consent form. A standardized clinical assessment was performed using the Unified Parkinson Disease Rating Scale (UPDRS) (Perlmutter, 2009) and the Hoehn & Yahr staging scale (Goetz et al., 2004). Pain and cognition were also assessed using the PainDetect (Freynhagen et al., 2006) and MoCa test (Berardelli et al., 2013).

The PainDetect questionnaire was applied to exclude specific pain conditions, and the MoCa test assured that the patient was able to understand the study procedures and was capable of giving informed consent to participate.

The following day, the patient arrived at the outpatient clinic of the Department for Neurology at Copenhagen University Bispebjerg Hospital in the morning between 7 and 9 am. The participants were required to fast and to stop their anti‐Parkinson medication 12 h before arrival. This corresponds to three times of the half‐life for levodopa. Motor symptoms in the pragmatic OFF‐medication state were assessed using the motor section of the UPDRS rating scale followed by standardized AMG measurements. The subjects then took an oral dose of levodopa (200 mg) and received a breakfast half an hour after oral drug intake. The second and third UPDRS motor section III assessments were carried out exactly one and 3 h after levodopa intake along with a standardized AMG measurement during the ON‐medication state.

Healthy subjects were recruited and informed about the protocol for the study. When they reported they wished to participate, and having given informed consent, they were invited for one visit only a couple of days later. Like in the patient group, we first assessed motor function using the motor UPDRS rating scale, and this was followed by a standardized AMG measurement. The healthy subjects had completed the PainDetect questionnaire prior to their visit to assure that they did not suffer from any pain conditions which could affect their motor function.

### 2.5 Acoustic myography

AMG was performed in real time using a mobile CURO unit featuring up to four acoustic sensors (CURO‐Diagnostics ApS) connected to an iPadAir (Apple Inc.). The acoustic sensors had a natural unfiltered frequency recording range of 0.5–20 ± 0.5 kHz. Acoustic muscle activity was recorded with the sensor placed over the central part of the muscle belly. The muscles selected for measurement in this study were ones that had previously been measured in AMG studies of healthy subjects and patients, were easily accessed and which were deemed relevant from the perspective of a clinical examination. The acoustic sensors were coated with ultrasound gel (EKO Gel, EkkoMed, Holstebro, Denmark) and taped to the skin using a flexible bandage (Mediplast AB, Malmö, Sweden). A sensor with a diameter of 5 cm was used for *biceps, triceps*, and *extensor carpi radialis longus muscle*. A sensor with a diameter of 2 cm was used *for adductor policis muscle*. Sampling rate was 2000 Hz per channel without. No band‐pass filtering was applied, and the signal was amplified (6 dB). The digitized data were stored on an iPadAir (Apple Inc.) as WAV file for further processing.

### 2.6 Motor testing procedure

The upper PD most affected extremity was measured following the procedures listed below.

1. Bending and stretching of the elbow joint (*m. biceps brachii, m. triceps brachii*)—passive and active. The range of movement was 120°, and the repeat was 10 times with counting such that each bend‐stretch lasted for approximately 2 s.
2. Wrist movement bending the hand backward and back toward the horizontal (*m. extensor carpii radialis longus*)—passive and active. The range of movement was 45° up to extension and 45° down to flexion, and the repeat was 10 times with counting such that each bend‐stretch was 2 s.
3. Distal part of the thumb touching the distal part of the little finger (both to the distal interphalangeal joint) 10 times (*m. adductor policis*)—active. The range of movement was up to 90°, and the time for one movement 2 s.

All the aforementioned movements were performed identically for PD and HS.

### 2.7 Data handling

**FIGURE 1:**
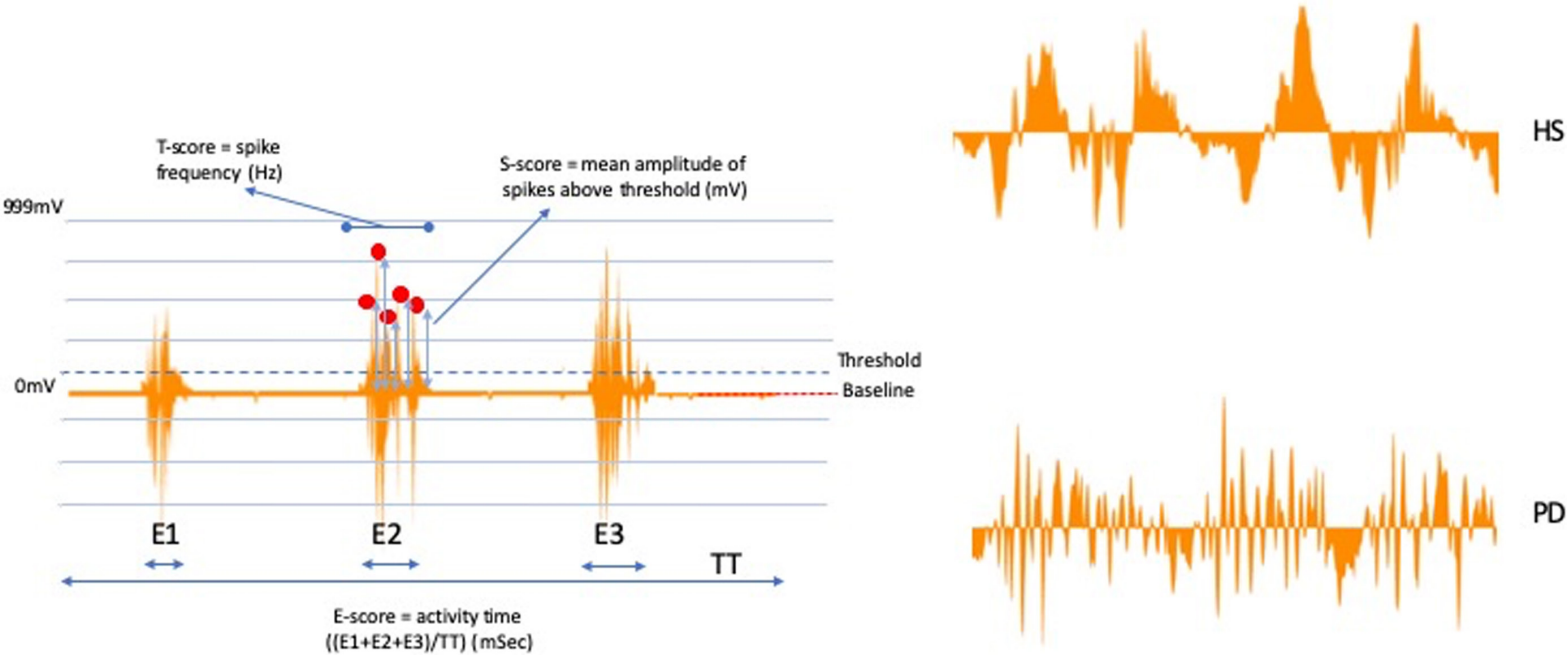
Acoustic myography (AMG) signal and measures of motor activity. The duration of AMG activity per movement was used as measure of motor efficiency (E‐score, ms). Spatial summation of motor activity was reflected by the S‐score (mV) reflecting the area under the curve of the AMG signal above a threshold value. Temporal summation was assessed using the spike frequency (Hz), indicating the number of activity peaks during the activity time. TT = total recording time where the sum of E1, E2, and E3 are expressed in relation to TT and represent muscle activity. The red line represents baseline (0 mV). The blue dotted line represents the threshold (typically threshold is set to 7% of the maximum amplitude scale) spikes above threshold being analyzed for amplitude and frequency (red • = AMG signals that have been analyzed). Top right‐hand panel (HS) is a typical recording from a healthy subject, while the lower right‐hand panel (PD) is from a PD patient.

The AMG data were analyzed using the CURO data handling program (CURO‐Diagnostics ApS), giving the ESTi™‐score with its individual components Efficiency/coordination (E‐score) and fiber recruitment including both Temporal (T‐score) and Spatial (S‐score) summation, which are all mean values over a defined time interval (Harrison, 2018) (for details, see Figure 1). This time interval was approximately 20 s in duration, and all the data within each frame were analyzed. The E‐score measures the periods of muscle contraction relative to an overall recording period (typically 30 s), hence it can be seen as representing muscle efficiency or the degree of coordination (Smith et al., 2005). The score value for each parameter is between 10 and 0. When looking at changes in movement or load, a difference between means showing a *p*‐value >0.05 was considered to be nonsignificant (NS).

The UPDRS scores were calculated for each part of this assessment tool, as well as a total score. PainDetect was only used to make certain that pain was in the normal range, so as not to affect muscle use.

The S‐ and T‐scores were analyzed to identify any changes that might occur with PD compared with HS. This analysis examined the ratio between the S‐ (spatial summation) and T‐(temporal summation) scores in both the PD subjects and HS. This ratio was obtained by dividing the S‐score by the T‐score, to give either a value above or below one.

### 2.8 Statistical analysis

The data were initially tested for a normal distribution using Column Analyses and Normality in Prism (GraphPad InStat 3 for Mac [Version 3.0b, 2003; GraphPad Inc.]). Differences between means were tested for statistical significance using an unpaired adjusted two‐tailed *t*‐test with a check for possible type 1 errors. Further statistical procedures were also applied, namely an ANOVA with Tukey´s multiple comparison tests. Differences between means with an adjusted *p*‐value <0.05 were considered significant. Values are presented as the mean ± SD in tables and mean ± SEM in graphs.

## 3 RESULTS

### 3.1 Patients

Thirteen male and seven female PD patients were tested. The mean age was 68.5 ± 7.3 years. See Table 1 for a full description of the patient population.

**TABLE 1:** Patient characteristics.

Age (yrs)	Sex	Disease duration	LEDD	MoCA	UPDRS total at home^a^	UPDRS section III at home^a^	UPDRS motor section in “of”	UPDRS section III 1 h after levodopa intake	UPDRS section III 3 h after levodopa intake	Hoehn and Yahr
67	F	4	380	29	4	2	5	2	0	1
73	F	2	450	26	29	16	21	14	12	2
65	M	5	450	29	18	10	10	7	11	2
60	M	6	700	26	38	25	26	15	19	2
67	M	7	665	27	29	17	19	16	17	2
68	M	6	870	26	29	18	20	15	18	2
79	M	3	400	28	12	4	8	6	6	1
74	M	4	940	25	35	22	24	15	15	2
70	F	9	800	25	24	9	21	11	15	2
67	F	8	1107	28	8	3	9	2	2	2
74	M	5	450	24	34	22	19	13	12	2
51	F	10	405	28	4	1	8	3	3	1
84	M	6	450	28	27	18	19	9	14	2
61	M	2	120	25	13	9	11	7	3	1
62	F	1	300	29	15	11	12	7	7	1
67	M	5	920	23	30	12	17	14	11	2
69	M	5	694	26	19	6	9	4	4	1
76	M	6	800	26	27	16	18	8	11	2
73	M	7	300	30	27	19	20	11	14	1.5
64	F	6	632	25	42	22	26	10	6	2.5
68.5 ± 7.3	35%F	5.3 ± 2.3	591.6 ± 259.3	26.6 ± 1.9	23.2 ± 11	13.1 ± 7.4	16.1 ± 6.5	9.4 ± 4.5	10.5 ± 5.6	Median 2.0, 95% CI 1.4 to 1.9

^a^ Measurements taken at the patient's home by MC and EB in the medicine on state. UPDRS performed by MC.

### 3.2 Clinical assessment with unified Parkinson's disease rating scale (UPDRS)

**FIGURE 2:**
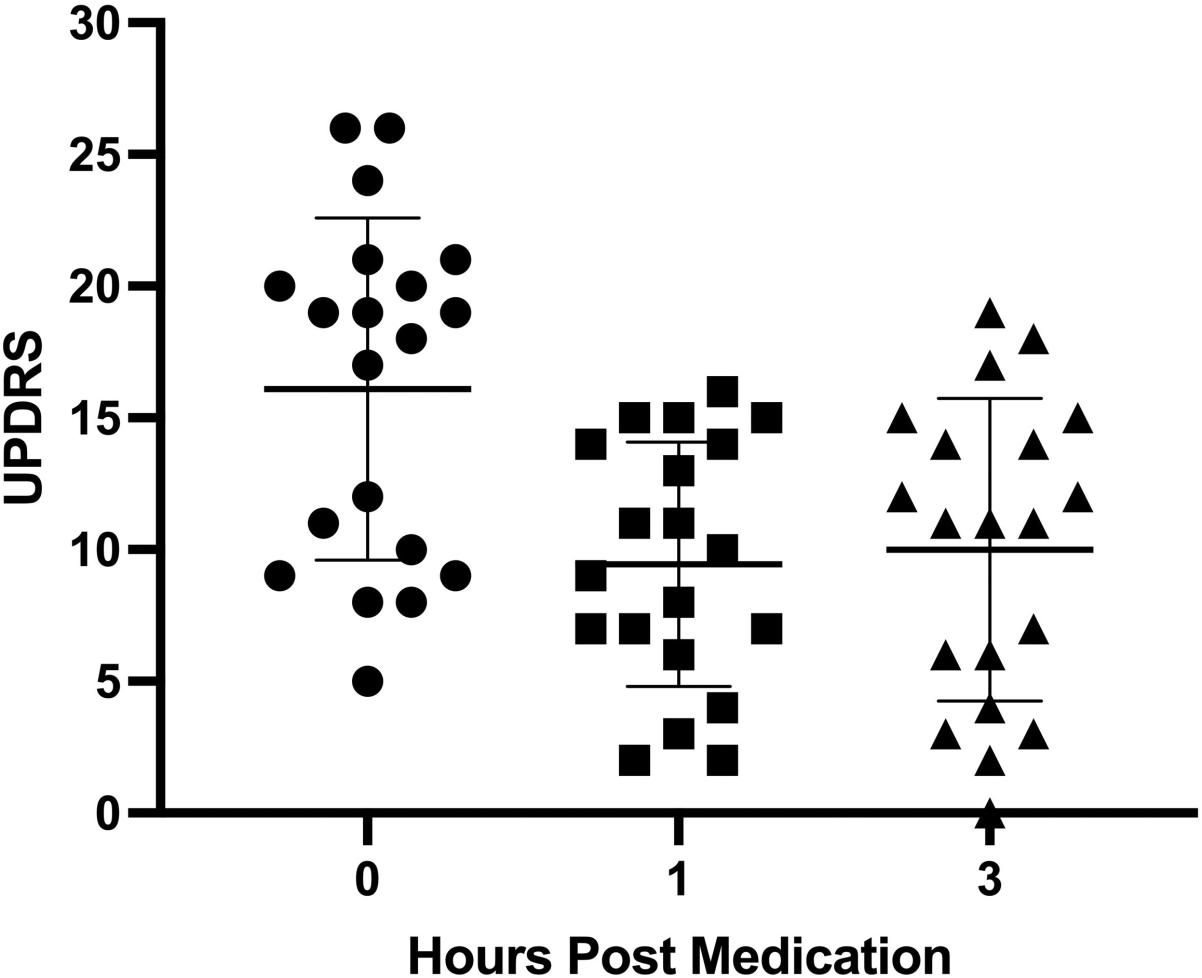
Unified Parkinson Disease Rating Scale (UPDRS) score dependence on levodopa intake. The number of subjects in each group (column) was 20. Data were tested for a normal distribution and normality, after which statistical significance was determined using an unpaired adjusted two‐tailed *t*‐test and an ANOVA with Tukey's multiple comparison. The individual values are presented in this dot plot, with mean as a horizontal line and SD values as vertical lines with bars; the Mean ± SD values for the three columns are ● off medication: 16.1 ± 6.3, ■ 1‐h postmedication: 9.4 ± 4.5, and ▲ 3 h post medication: 10.0 ± 5.6, respectively. Adjusted p‐values given.

UPDRS (Perlmutter, 2009) is a frequently used assessment scale for clinical assessment of Parkinson's patients. The results for the UPDRS are shown in Figure 2. Our data clearly demonstrates that removing the 20 patients from their medication had a dramatic and statistically significant effect on their UPDRS score when comparing this to scores at one and 3 h after re‐medication, respectively.

### 3.3 Acoustic myography

**FIGURE 3:**
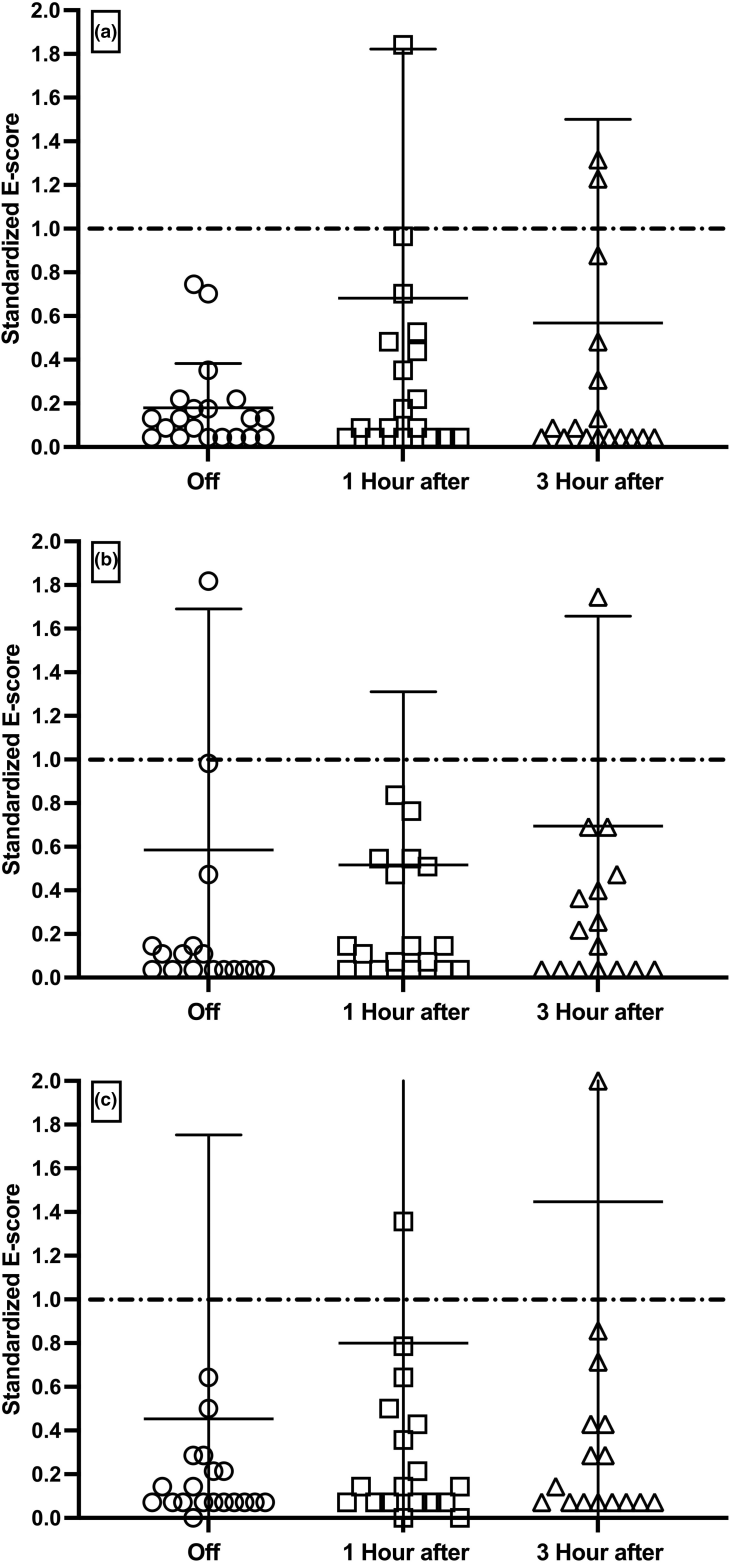
E‐score development following levodopa administration for *m. biceps* (Upper panel: A), *m. triceps* (Middle panel: B), *m. extensor carpi radialis longus* (Lower panel: C), during active movement. E‐score values were standardized to the mean of the HS value. ○ is off medication, □ is 1‐h post‐medication, and ᴧ is 3 h postmedication. The mean HS value is indicated in the figures as a dotted line, which serves as the standard value to which all the other data were subsequently compared. Therefore, the mean HS value has been set to 1.0 and the actual mean values and SD are as follows; Biceps 2.2 ± 2.8 ms, Triceps 2.7 ± 2.8 ms and ECRL 1.4 ± 1.5 ms. The individual values are presented in this dot plot, with mean as a horizontal line and SD values as vertical lines with bars. Data were tested for a normal distribution and normality, after which statistical significance was determined using an unpaired adjusted two‐tailed *t*‐test and an ANOVA with Tukey´s multiple comparison. The Mean ± SD values for the three columns and three graphs were 0.4 ± 0.4, 1.5 ± 2.5, 1.3 ± 2.0 and 1.6 ± 2.9, 1.4 ± 2.1 and 1.9 ± 2.5, and 0.6 ± 1.8, 1.2 ± 2.3, 2.0 ± 3.3, respectively. Statistically adjusted *p*‐values are given.

The results for active movement of *m. biceps* showed a statistically significant (*p* = 0.009) effect on the E‐score, when removing the 20 subjects from their medication, as compared to equivalent values for matched healthy subjects (see Figure 3). Once medication was reinstated, the E‐score improved and was not found to be statistically different from that of the healthy subjects. For passive movement, a significant effect was noted for both the E‐score and the S‐score compared with HS (see Table 2; *m. biceps brachii* and *m. extensor carpi radialis longus*). No significant effect was noted for *m. triceps brachii* in PD compared with HS for either active or passive movements.

**TABLE 2:** Mean values of E, S, and T for both active and passive movement for PD and HS for active and passive movements in *m. biceps brachii*, *m. triceps brachii*, and *m. extensor radialis carpi longus*.

	PD *m. biceps brachii*	HS *m. biceps brachii*
	Passive 0	Passive 0
Mean E‐score	0.91 ± 1.21^b^	2.14 ± 1.91^b^
Mean S‐score	3.74 ± 3.30^c^	6.68 ± 3.12^c^
Mean T‐score	4.52 ± 1.72	3.34 ± 1.98
	Active 0	Active 0
Mean E‐score	0.41 ± 0.46^a^	2.13 ± 2.76^a^
Mean S‐score	3.43 ± 3.30	5.36 ± 3.31
Mean T‐score	4.95 ± 2.46	3.83 ± 2.18

	PD *m. triceps brachii*	HS *m. triceps brachii*
	Passive 0	Passive 0
Mean E‐score	2.30 ± 3.13	2.73 ± 2.70
Mean S‐score	5.38 ± 3.67	6.41 ± 2.96
Mean T‐score	4.50 ± 1.75	3.73 ± 2.99
	Active 0	Active 0
Mean E‐score	1.61 ± 3.03	2.73 ± 2.90
Mean S‐score	4.76 ± 3.43	6.17 ± 3.03
Mean T‐score	4.59 ± 1.83	4.13 ± 3.20

	PD *m. extensor carpi radialis longus*	HS *m. extensor carpi radialis longus*
	Passive 0	Passive 0
Mean E‐score	0.58 ± 0.73^d^	3.64 ± 3.06^d^
Mean S‐score	4.94 ± 2.23^e^	7.45 ± 2.79^e^
Mean T‐score	4.11 ± 2.03	4.54 ± 2.97
	Active 0	Active 0
Mean E‐score	0.66 ± 1.86	1.50 ± 1.59
Mean S‐score	2.48 ± 2.16^f^	6.07 ± 3.51^f^
Mean T‐score	6.30 ± 1.89^g^	3.65 ± 1.94^g^

*Note*: ^a^E—PD Ba0 versus HS Ba0 = 0.0093; ^b^E—PD Bp0 versus HS Bp0 = 0.020; ^c^S—PD Bp0 versus HS Bp0 = 0.006; ^d^E—PD ECRLp0 versus HS ECRLp0 = 0.0002; ^e^S—PD ECRLp0 versus HS ECRLp0 = 0.0049; ^f^S–PD ECRLa0 versus HS ECRLa0 = 0.0006; ^g^T—PD ECRLa0 versus HS ECRLa0 = 0.0001.

With regard to *m. extensor carpi radialis longus*, there was a significant difference in the PD passive E‐score and S‐score compared with HS, and in the active S‐score and T‐score again in PD compared with HS (see Table 2).

For the fourth muscle measured in this study, namely *m. adductor policis*, AMG data showed inconsistent values due to the fact that the sensor became intermittently detached from the surface of the muscle body during activation of this muscle group, and these data are not shown for this reason.

When comparing the E‐score for m. *biceps brachii* in PD with the UPDRS score at the three time points (without medication, 1 and 3 h after medication), there was a clear correlation, which was observed as being a higher E‐score and lower UPDRS score during medication, both in passive and active flexion/extension of the elbow.

**FIGURE 4:**
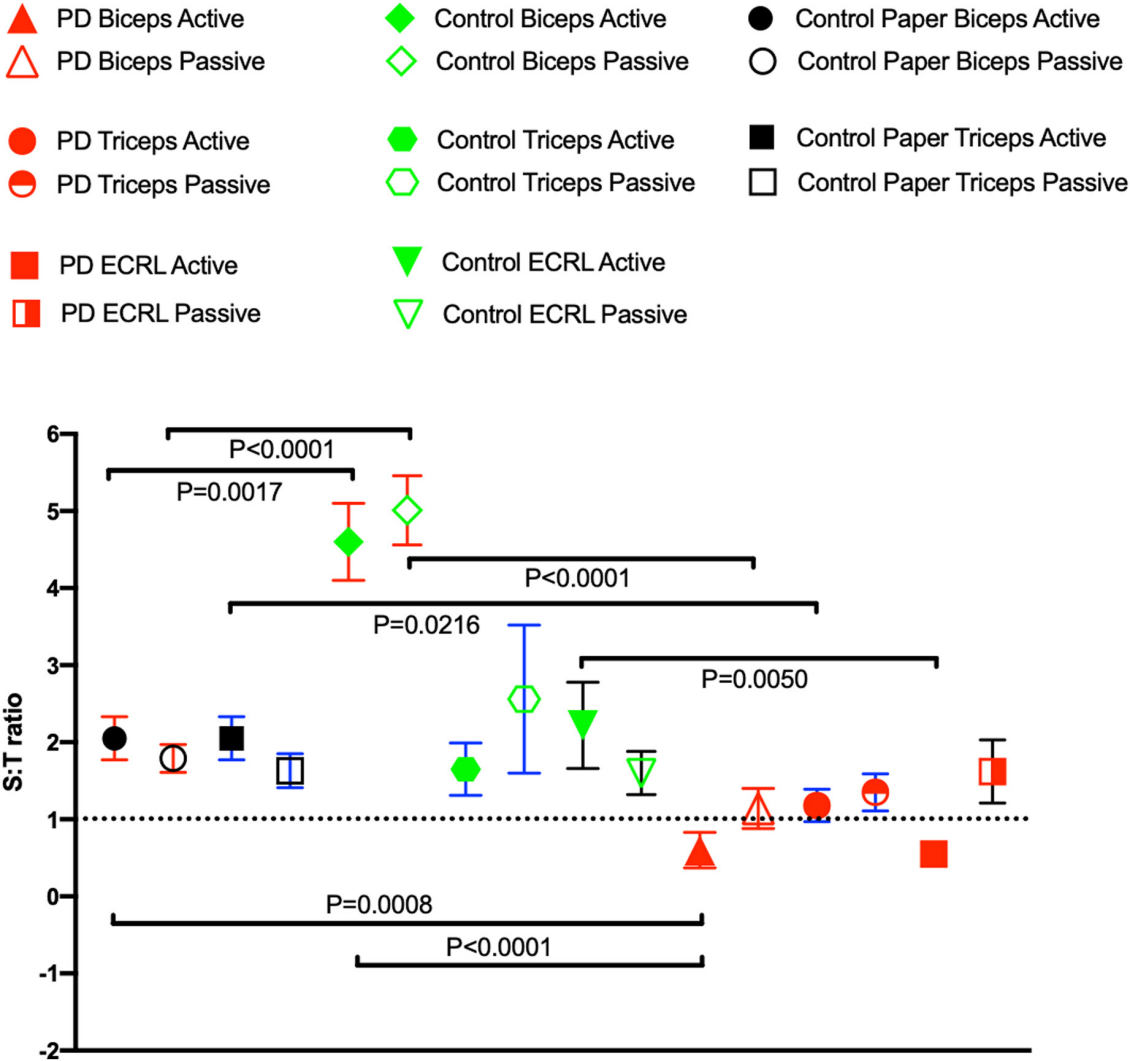
S:T‐score ratio values for PD (red symbols) and HS (green symbols) muscles compared with reference values for the 60–69‐year‐old group in (black symbols) (Bartels, Ahmed, et al., 2020). Among the HS group, three subjects showed a S:T that was 10‐fold higher than the rest, and these are not included in the figure as they would affect the clarity of the results. This selective removal does not, however, change the general impact of the results. X‐axis represents examined muscles. Y‐axis represents the S:T‐score calculated as the mean of the spatial and temporal summation [S‐score (mV) and T‐score (Hz)]—shown to be highly correlated with muscle force production (Claudel et al., 2018). Data were tested for a normal distribution and normality, after which statistical significance was determined using an unpaired adjusted two‐tailed *t*‐test and an ANOVA with Tukey´s multiple comparison. The means are of 20 subjects except for the HS group which comprises 17 and for the 60–69‐year‐old group (Bartels, Ahmed, et al., 2020) where there were 10 subjects. Due to the varying number of subjects in these groups, SEM has been applied rather than SD. Statistically adjusted p‐values are given.

As can be seen in Table 2 and in Figure 4 for all muscles, the T‐score is always lower than the S‐score for arm bending in healthy subjects, resulting in a S:T ratio that is >1. Put another way, temporal summation > spatial summation. It is likewise interesting that the S:T ratio remains clearly above the value of one for all muscles for HS. For PD, however, the S:T ratio is found to be lower than that for the healthy subjects, and generally around one. While for two muscles in particular (*m. biceps brachii* and *m. extensor carpi radialis longus*), active arm bending results in a change in the S:T ratio such that the value becomes <1, in other words, spatial summation > temporal summation.

Note that the S:T ratio is well above one for the HS muscles while for PD this ratio lingers around 1. Values for the healthy 60–69‐year‐old reference group muscles were also found to be above one. This means that values for the S:T ratio that are above one indicate muscle activity that principally involves less active muscle fibers but a higher firing rate, while a value below one indicates relatively more active fibers during movement but with a lower firing rate.

## 4 DISCUSSION

**FIGURE 5:**
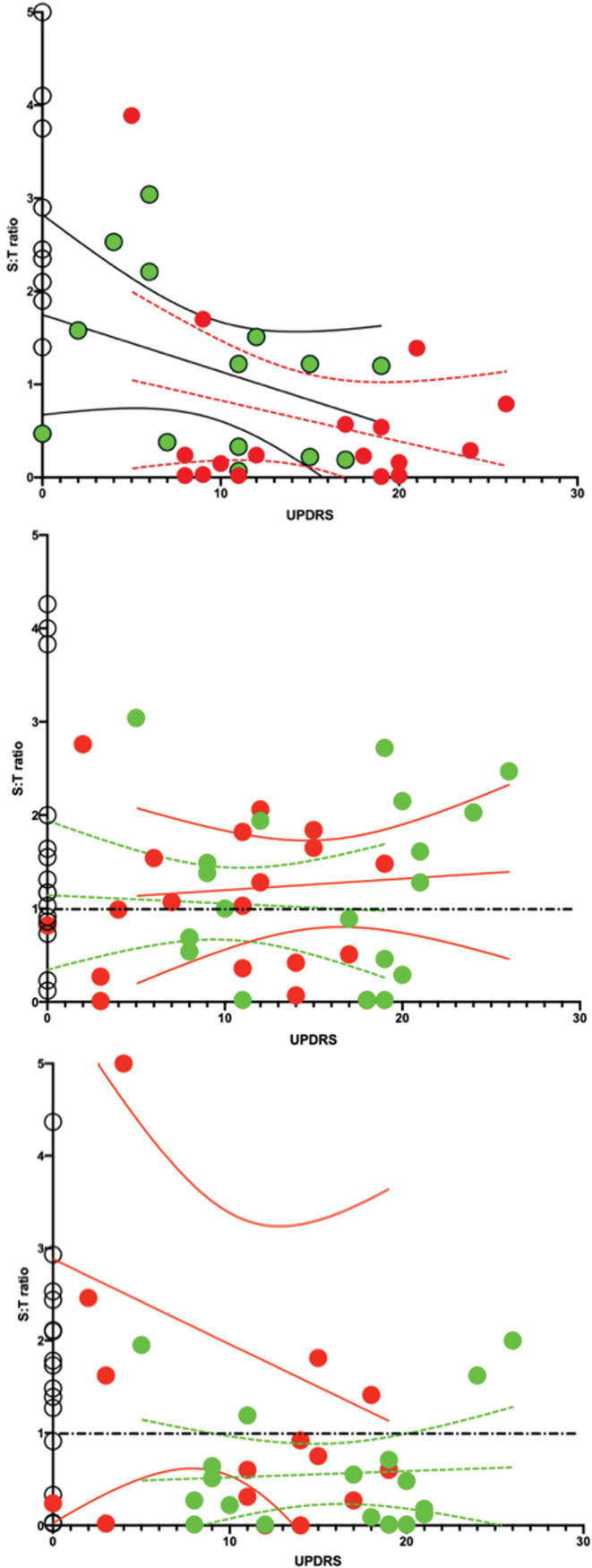
S:T ratio as a function of the total Unified Parkinson Disease Rating Scale (UPDRS) score for the three muscles measured. Panel (A) *m. biceps*, panel (B) *m. triceps* and panel (C) is for *m. extensor carpi radialis longus*. Notice open circles represent HS and red circles represent PD subjects off medication and green circles represent PD subjects 3 h after return to medication. The straight lines represent the mean and the bent lines represent the 95% percentile. The correlation coefficient *r* and the sample size were as follows; (A) PD0 *r* = 0.28, *n* = 19 and PD3 *r* = 0.37, *n* = 19; (B) PD0 *r* = 0.08, *n* = 19 and PD3 *r* = 0.06, *n* = 19; (C) PD0 *r* = 0.06, *n* = 19, and PD3 *r* = 0.2, *n* = 19. In addition, the mean values for the HS groups which are standardized to S:T ration = 1.0 for panels (A), (B), and (C) are 4.6, 1.7, and 5.6, respectively.

This study set out to test AMG as a new easily applicable assessment tool for Parkinson's disease, expecting changes in the AMG scores which could characterize Parkinson's disease when compared to values for HS. The most important finding of this study is that the S:T ratio changes from being principally temporal summation (higher motor unit firing rate) in HS, for the movements studied here, to being primarily spatial summation (more motor units active) with PD in *m. biceps brachii* and *m. extensor carpi radialis longus*. An additional finding of clinical importance is that the S:T‐score was found to correlate to the total UPDRS score in these muscles (see Figure 5).

Looking at passive and active elbow flexion and extension, *m. biceps brachii* in PD in a medical off state showed a lower E‐score (poorer coordination) than in HS in both passive and active movements. Furthermore, the S‐score was also lower in PD than in HS during both passive and active movement. Interestingly, m. *triceps brachii* did not show any differences in AMG scores between PD and HS during these elbow movements. When looking at AMG scores during flexion and extension of the wrist joint, PD in a medical off state showed lower E‐ and S‐scores during passive movements, and lower S‐ and higher T‐scores during active movement when compared to HS. Following levodopa intake, the differences between PD and HS disappeared. The changes in E‐score seen in *m. biceps brachii* and *m. extensor carpi radialis longus* were, as would be expected, inversely related to the total UPDRS score.

Looking at the S:T ratio, PD in a medical off state showed a clear difference from HS for the *m. biceps brachii* and *m. extensor carpi radialis longus* both during active movement only. The ratio in HS was for all three muscles well above one, while for PD, the S:T ratios lingered around 1.

Changes in muscle function in PD subjects upon return to medication have been monitored with surface electromyography (sEMG) (Cantello, 1995). Levin et al. (2009) thought it important that a physician should be able to activate a patient's arm muscles by moving their arm for them and in this way generate muscle stretch. Their findings revealed that the sEMG pattern obtained during such passive movements showed stretch‐related activity, which proved to be statistically significant between PD and HS, but only during the stretch phase and not during the phase of release (Levin et al., 2009). It was these results that led Levin et al. (2009) to conclude “*that rigidity in PD subjects is caused by sEMG or at least by sEMG‐associated alterations of central origin that are only present during muscle stretch*,” Of course, such a conclusion runs against the belief that rigidity in PD subjects originates peripherally and is the result of changes in muscle structure (Meara & Cody, 1992).

The data of Cantello (1995), Smith et al. (2005) support those of the present study, not only showing that the UPDRS level declines with a return to medication, but also that muscle‐specific changes with sEMG inversely mirror the UPDRS change. Furthermore, myometry measurements likewise confirm that the UPDRS change is linked to a change in tension (Marusiak et al., 2010). However, sEMG represents a combination of electrical signals from both the nerve and muscle (Harrison, 2018), making assessment of changes somewhat imprecise in terms of their origin, while AMG measures solely muscle contraction.

**FIGURE 6:**
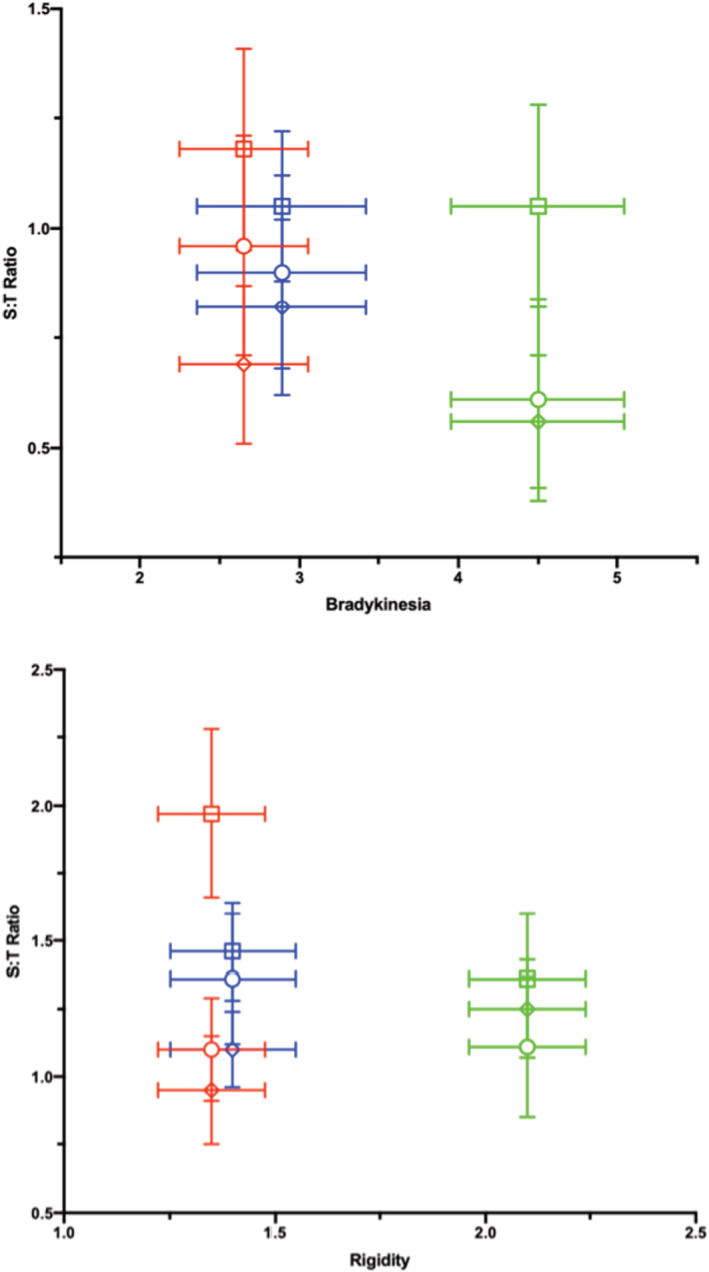
Bradykinesia represents what happens in the active muscle while rigidity is measured during passive movement. Looking at the Unified Parkinson Disease Rating Scale (UPDRS) sub‐scores for bradykinesia and for rigidity, no significant correlation to the S:T‐score (correlating closely with muscle force [Claudel et al., 2018]) was found for the three muscles measured although a return to medication reduced both scores. Green symbols = time 0, red symbols = time 1, and blue symbols = time 3. Round symbols = *m. biceps*, square symbols = *m. triceps* and diamond symbols = *m. extensor carpi radialis longus*. These two figures reveal how a return to medication reduces both scores and there appears to be a tendency toward an increase in the S:T ratio. Data which are presented as the means ± SD (*n* = 20) were tested for a normal distribution and normality, after which statistical significance was determined using an unpaired adjusted two‐tailed *t*‐test and an ANOVA with Tukey´s multiple comparison.

A more detailed analysis of the UPDRS subscales for bradykinesia and for rigidity can be seen in Figure 6. It was found that a return to medication reduced both scores, as might be expected, but that no significant correlation was detected with the S:T ratio.

The UPDRS scale assesses various aspects of control of muscle activity and coordination (Perlmutter, 2009). Looking at the subscales for bradykinesia (active movement) and rigidity (passive muscle) is interesting in the muscles used for daily activities, both being important issues in PD. With only 20 patients included in this study, however, the correlation shows trends. The clear correlation of the S:T ratio to total UPDRS implicates that the sum of the scores from the various UPDRS subscales gives a fuller picture of the muscle challenges in PD.

Marusiak et al. (2018) concluded that rigidity in PD muscles compared with healthy subjects was related to changes in the viscoelastic properties of muscles, since their electromyographic and mechanomyographic data did not show differences between the two groups. The present study, however, has been able to detect acoustic myography changes with a return to medication that was significant.

In a recent study by Annweiler et al. (2013), it was shown using magnetic resonance spectroscopy (MRS) that older adults with cognitive impairment show not only a decrease in motor cortex volume but changes in key metabolite ratios too, all of which are associated with a slower gait velocity and a greater stride time variability. Specifically, these authors found that a slower gait velocity was correlated with higher choline‐to‐creatine ratios in the brain (Annweiler et al., 2013). Subjects with PD and also Alzheimer´s disease have higher stride time variability compared with HS (Beauchet et al., 2008), supporting the idea that regular gait is associated with neural function, perhaps even at the molecular level (Annweiler et al., 2013). Thus, one could speculate that changes in motor cortex volume and metabolite ratios with PD results in a decreased temporal summation capability, leading to a compensatory change toward increased spatial summation in key muscles, something that is measurable in PD muscles as an increased tonus or rigidity as a result of a greater percentage of active fibers.

The difference seen in this study between *m. biceps brachii* and *m. triceps brachii* in the PD patients may indicate the underlying neural innervation difference between flexor and extensor (Anderson & Bordoni, 2020). This is further supported by the finding of an improvement in resting elbow joint angle following medication (Marusiak et al., 2018), supporting the idea that rigidity is present to a higher degree in flexors than in extensor muscles and possibly due to a higher resting tone of the flexor muscles cf extensors (Andrews et al., 1972). It should of course be noted that the Marusiak study (2018) (McLellan, 1973) involved static measurements, while the present study was undertaken during periods of active muscle movement. Another explanation could simply be the position of the upper extremity in relation to the movement measured, thereby being an effect of gravity and proprioception (Anderson & Bordoni, 2020; Apker et al., 2011).

The difference seen between *m. biceps brachii* and *m. triceps brachii* may very well be due to the difference in activation of these two major forearm muscles. The clear AMG pattern seen for *m. biceps brachii* may indicate an underlying impaired function in more than the pyramidal pathway and there is evidence that there may also be extra‐pyramidal involvement (Berardelli et al., 1983; Moustafa et al., 2016; Sciutti et al., 2012). A significant difference in the E‐score was found between healthy subjects and PD subjects in the extensor *m. extensor carpi radialis longus*, for passive movement. This would, as for *m. biceps brachii*, indicate a slower “switch off” of the muscle fibers, thereby creating a higher resting muscle tone, which would be detected as rigidity.

Acoustic myography as an assessor of muscle function has the advantage of being noninvasive and easy to use, and it does not involve pain or discomfort. It also lends itself to distance monitoring which could be advantageous for both patients and clinicians alike, allowing for home monitoring during daily activities (Bartels, Olsen, et al., 2020; Pingel et al., 2019). Compared with sEMG, the analysis of data is more straight forward and with less question of interference from other signals (Harrison, 2018). From our data, measuring the S:T ratio for m. *biceps brachii* and m*. extensor carpi radialis longus* during active movement may prove a useful tool in diagnosing PD, since a ratio just above or below 1 will indicate possible PD. Looking at the E‐score in m. *biceps brachii* could be a possible way to measure during medication adjustment, especially since other methods like myometry only measure muscle tension parameters (tone & stiffness) (Ferreira‐sánchez et al., 2020) and cannot provide details concerning coordination of muscle contractions as well as both spatial and temporal summation during muscle use (Harrison, 2018).

Measuring the S:T‐score in *m. biceps brachii* appears to be a promising method to assess early PD by a simple active flexion and extension of the elbow. Measuring the passive and active E‐score during the same movement and from the same muscle will, furthermore, be a useful tool when adjusting medication in PD. The correlation to total UPDRS for these parameters indicates that AMG may be a new assessment tool for PD. The changes found in the UPDRS in the present study further confirm the finding that the E‐score for active *m. biceps brachii* provides a measure of the effect of medication and may be the missing tool when fine‐tuning the dose for individual PD subjects.

## 5 CONCLUSIONS

This study has revealed that the ratio between the S‐ and T‐score (S:T) may prove an important indicator of early disease onset affecting muscle control with PD, as well as a means of monitoring and following treatment. The E‐score during active elbow flexion/extension could also be a quick and noninvasive assessment tool in PD. AMG could, furthermore, be used for home monitoring by the subjects themselves.

## AUTHOR CONTRIBUTIONS

M. Celicanin, E. M. Bartels, A. P. Harrison, Løkkegård, B. Danneskiold‐Samsøe, and H. R. Siebner contributed to the conception and design of the work. M. Celicanin and E. M. Bartels performed the measurements. M. Celicanin, E. M. Bartels, J. Kvistgaard Olsen, and A. P. Harrison analyzed the data. M. Celicanin drafted the initial manuscript. All authors have read, revised, and approved the manuscript.

## FUNDING INFORMATION

Hartwig R. Siebner holds a 5‐year professorship in precision medicine at the Faculty of Health Sciences and Medicine, University of Copenhagen which is sponsored by the Lundbeck Foundation (Grant Nr. R186‐2015‐2138). The Parker Institute was supported by core funding from the Oak Foundation (Ocay‐13‐309). The project was supported by Erna Hamiltons Fond.

## CONFLICT OF INTEREST STATEMENT

APH is in the process of establishing a company to produce and market the Acoustic MyoGraphy (CURO‐Diagnostics ApS). Hartwig R. Siebner has received honoraria as speaker from Sanofi Genzyme, Denmark, and Novartis, Denmark, as consultant from Sanofi Genzyme, Denmark, Lophora, Denmark, and Lundbeck AS, Denmark, and as editor‐in‐chief (Neuroimage Clinical) and senior editor (NeuroImage) from Elsevier Publishers, Amsterdam, The Netherlands. He has received royalties as book editor from Springer Publishers, Stuttgart, Germany, and from Gyldendal Publishers, Copenhagen, Denmark.

## ETHICS STATEMENT

The study followed the guidelines set by the Helsinki Declaration 2013 (https://www.wma.net/policies‐post/wma‐declaration‐of‐helsinki‐ethical‐principles‐for‐medical‐research‐involving‐human‐subjects/), and all subjects gave an informed written consent prior to participating in the study. The study was approved by the Capital Region of Denmark's Ethics Committee (No. H‐17021637) and was registered at the Danish Data Protection Agency. All data were collected and handled according to The Act on Processing of Personal Data (Act No. 429 of May 31, 2000, with amendments (latest 2018)).
